# Transcriptomic Analysis Identifies Differentially Expressed Genes Associated with Vascular Cuffing and Chronic Inflammation Mediating Early Thrombosis in Arteriovenous Fistula

**DOI:** 10.3390/biomedicines10020433

**Published:** 2022-02-13

**Authors:** Vikrant Rai, Devendra K. Agrawal

**Affiliations:** Department of Translational Research, Western University of Health Sciences, Pomona, CA 91766, USA; vrai@westernu.edu

**Keywords:** adventitial inflammation, arteriovenous fistula, chronic inflammation, early thrombosis, fibrosis, maturation, maturation failure, perivascular cuffing

## Abstract

Arteriovenous fistula (AVF) is vascular access created for hemodialysis in end-stage renal disease patients. AVF creation causes increased blood flow in the outflow vein with increased pressure. Increased blood flow, blood volume, and shear stress causes outward remodeling so that the outflow vein can withstand the increased pressure. Outward remodeling of the vein involved in AVF is necessary for AVF maturation, however, inward remodeling due to excessive neointimal hyperplasia (NIH) and chronic inflammation may end up with vessel thrombosis and AVF maturation failure. Early thrombosis of the vessel may be due to the luminal factors including NIH and chronic inflammation or due to chronic inflammation of the adventitial due to perivascular cuffing. Inflammation may either be due to an immune response to the vascular injury during AVF creation or injury to the surrounding muscles and fascia. Several studies have discussed the role of inflammation in vascular thrombosis due to intimal injury during AVF creation, but there is limited information on the role of inflammation due to surrounding factors like a muscle injury. The concept of perivascular cuffing has been reported in the nervous system, but there is no study of perivascular cuffing in AVF early thrombosis. We performed the bulk RNA sequencing of the femoral arterial tissue and contralateral arteries as we found thrombosed arteries after AVF creation. RNA sequencing revealed several significantly differentially expressed genes (DEGs) related to chronic inflammation and perivascular cuffing, including tripartite motif-containing protein 55 (TRIM55). Additionally, DEGs like myoblast determination protein 1 (MYOD1) increased after muscle injury and relates to skeletal muscle differentiation, and network analysis revealed regulation of various genes regulating inflammation via MYOD1. The findings of this study revealed multiple genes with increased expression in the AVF femoral artery and may provide potential therapeutic targets or biomarkers of early thrombosis in AVF maturation failure. Thus, not only the luminal factors but also the surrounding factors mediating vascular cuffing contribute to vessel thrombosis and AVF failure via early thrombosis, and targeting the key regulatory factors may have therapeutic potential.

## 1. Introduction

Arteriovenous fistula (AVF), an abnormal connection between an artery and a vein, is vascular access created for long-term hemodialysis in end-stage renal disease (ESRD) patients. In AVF, blood flows directly from an artery into a vein bypassing capillaries and subjecting the outflow vein to increased blood pressure and shear stress [1]. The creation of AVF is associated with acute inflammation which is necessary for the wound healing and resolution phase but chronicity of inflammation results in thrombosis of the vessels involved in AVF and leads to AVF maturation failure. Thrombosed AVF with chronic inflammation is characterized by increased C-reactive protein (CRP), infiltration of the immune cells including neutrophils and macrophages, increased expression of vascular cell adhesion protein (VCAM)-1, interleukin (IL)-6, and tumor necrosis factor (TNF)-α, neoangiogenesis, neointimal hyperplasia (NIH), and atheromatous plaque formation [2,3,4,5]. Intimal injury while creating AVF may cause endothelial dysfunction and chronic inflammation leading to AVF maturation failure [6]. Additionally, inflammation within the vicinity of AVF either due to vessel injury increasing vascular leakage or due to inflammation in the surrounding tissues including muscles causing vascular cuffing may also contribute to vessel thrombosis and AVF maturation failure. Muscle injury, either mechanical or contusion, precipitates inflammation associated with increased infiltration of immune cells including neutrophils, macrophages, natural killer (NK) cells, B- and T-lymphocytes; the secretion of pro-inflammatory cytokines including TNF-α, IL-6, IL-8, IL-1β, IL-1α, macrophage inflammatory protein 1 alpha (MIP-1α), and monocyte chemoattractant protein (MCP)-1 and growth factors including granulocyte colony-stimulating factor (G-CSF), macrophage colony-stimulating factor (M-CSF), vascular endothelial growth factor (VEGF), hepatocyte growth factor (HGF), fibroblast growth factor (FGF), and platelet-derived growth factor (PDGF). 

Acute inflammation is necessary for the resolution and remodeling phase of inflammation; however, chronicity of inflammation leads to fibrosis [7,8,9]. Creation of AVF involving the femoral artery and vein induces muscle injury and non-resolving inflammation may lead to the fibrosis of the muscle and the surrounding tissue. After creation of AVF between the femoral artery and vein, the tissues are sutured in multiple layers within the muscles, subcutaneous tissues, and skin. Post-surgery, dissected tissues and surgical wound heal may induce acute inflammation, but chronicity of inflammation may cause fibrosis of the tissue surrounding AVF and thrombosis of the vessels. Based on this, we hypothesize that the chronicity of inflammation in the surrounding tissue precipitating vascular cuffing mediate thrombosis, stenosis, and fibrosis of the vessels [10] and the area surrounding AVF. This will lead to early thrombosis of the vessels and early AVF maturation failure. Thus, to investigate the effects of muscle injury during AVF creation, the presence of chronic inflammation, perivascular cuffing, and thrombosis and its association with early thrombosis of the vessels and to compare the transcriptomic profile of the contralateral control femoral artery (contralateral FA), untreated and treated femoral artery involved in AVF (AVF FA), we performed the bulk RNA sequencing of the control femoral artery and AVF tissue femoral artery treated with the inhibitors of triggering receptor expressed on myeloid cells-1 (TREM-1) and toll-like receptor-4 (TLR-4). The tissues were collected from the miniswine being used for another ongoing study in the lab to investigate the effect of TREM-1 and TLR-4 inhibition on AVF maturation. The effects of TREM-1 and TLR-4 inhibition on early vessel thrombosis was under investigation because pro-inflammatory mediators TREM-1 and TLR-4 play a critical role in atheromatous plaque formation, atherosclerosis, and vessel stenosis [11,12,13,14]. The aim was to compare the gene expression profile between the groups to elucidate differentially expressed genes (DEGs) related to chronic inflammation. 

## 2. Materials and Methods

### 2.1. Animal Model, AVF Creation, and Tissue Collection

For this study, female Yucatan miniswine, four to seven months old and weighing between 20–30 kg, purchased from Premier Bio-resources (Cotati Ramona, CA, USA) randomly divided into two experimental groups were used to create AVF fistula involving the right femoral artery (FA) and femoral vein (FV). The contralateral FA and FV were used as biological controls. Yucatan miniswine were housed in the vivarium of Western University of Health Sciences, Pomona, CA with 12 h light and dark cycle at a temperature range of 72–74 °F and fed with the Mini-Pig Grower Diet (Test Diet # 5801) and allowed to drink water ad libitum. Female pigs were used because of their less aggressive behavior and ease of handling compared to the males. All experiments involving the animals were performed as per National Institutes of Health and USDA guidelines for the care and use of experimental animals. The Institutional Animal Care and Use Committee (IACUC) at Western University of Health Sciences approved protocol No. R20IACUC038 for this study. 

For AVF creation, there were two experimental groups: (i) animals treated with LR-12 + TAK242, and (ii) animals treated with scrambled peptide and 30% ethanol (the vehicle for TAK-242). These animals were also used for another ongoing study in the lab to investigate the effect of inhibiting the triggering receptor expressed on myeloid cells-1 (TREM-1) and toll-like receptor-4 (TLR-4) on early thrombosis of the artery and fistula after creating AVF. Each experimental group consisted of three to four animals with similar body weights. For creating AVF, minipigs were first given a preanesthetic sedative injection of 2.5–5 mg/kg Telazol (a combination of tiletamine and zolazepam) and 1–2 mg/kg xylazine subcutaneously. After sedation, the animals were moved to the operating suite and intubated with an appropriately sized endotracheal tube, maintained on inhaled isoflurane in oxygen 1–3% and mechanical ventilation. After starting an intravenous ringer lactate (5 mL/kg/h) using the ear vein, the AVF was surgically created between FA and FV after preparing them for side-to-side anastomosis. For AVF creation, a 1 cm incision was made on the medial side of FA and FV opposing each other. AVF was created using a 6–0 proline to join FA and FV. LR-12 (>10^9^ particles in 1 mL), bolus TAK-242 (3 mg/kg dissolved in 30% ethanol), scrambled peptide, and vehicle control (30% ethanol) was injected at the AVF side in the lumen of FA and FV, and we waited for 5–10 min before closing the AVF. The wound was closed in layers suturing muscles, subcutaneous tissues, and skin in layers using 3–0 vicryl sutures. The swine were given a maintenance dose of TAK-242 once daily 0.1 mg/kg i.v. for six days and then weekly once for four weeks. During surgery, the level of sedation, percentage of isoflurane received, oxygen flow rate, heart rate, respiratory rate, mucous membrane color, presence or absence of withdrawal reflex, and body temperature were maintained every 15 min by a veterinary technician. Post-surgery, the swine were given 1 g of cefazolin prophylactically and buprenorphine for pain. The sedation was reversed with flumazenil 0.01 mg/kg. The swine were monitored post-surgically until they were on their feet, and recovery parameters including consciousness level, recumbency, respiratory rate and character, and mucous membrane color were monitored every 1 h until the swine started walking.

After completing 12 weeks of AVF creation, the swine were sedated, and radiological assessments of FA, AVF, and FV using ultrasonogram (USG), angiography, and optical coherence tomography (OCT) were done. This was followed by euthanasia using intravenous administration of a single dose of euthanasia solution consisting of pentobarbital sodium (85 mg/kg) and phenytoin sodium (11 mg/kg) while the pigs were under anesthesia. Swine were observed for the absence of heartbeats and respiration for at least 10 min before tissue harvest. The tissue of interest including AVF involved FA and FV, tissue around the fistula, and contralateral FA and FV were harvested after dissecting the groin area. The tissues were harvested for histomorphological studies in 10% formalin, for RT-PCR and sequencing studies in RNA later, and for protein isolation at 4 °C and stored at −80 °C. For histomorphological studies, the harvested tissues were processed in a tissue processor and paraffin embedded. Five µm thin sections using a tungsten carbide knife (LeicaTM, Germany) in a Leica RM2265 rotary microtome (LeicaTM, Germany) were sectioned and attached to glass slides for histology.

### 2.2. Radiological Assessment of the AVF

To assess the diameter of the FA and FV, flow velocities and flow volume in the FA at baseline, a preoperative color Doppler ultrasound (Phillips EPIQ-7 US system) was done before creating AVF. After 12 weeks, to evaluate the AVF patency, FA flow and diameter, outflow vein diameter, and the flow velocity in the vein, a postoperative USG of the AVF, FA, and FV at the AV anastomosis site was done before sacrificing the miniswine. Each AVF site was evaluated for the peak systolic and end-diastolic velocity and blood flow at different locations in the artery and vein of the AVF, including the anastomosis site. Color Doppler ultrasound was followed by femoral angiography using a 7F (Concierge) guide catheter from Merit Medical USA through the carotid artery to assess the patency and blood flow in the fistula on the anastomosis site and the contralateral side FA for comparison. Briefly, angiography was performed by percutaneous needle puncture in the common carotid artery under ultrasound guidance and an angiography catheter was advanced from the carotid artery into the descending aorta, external iliac arteries to the FA of the AVF side as well as to the contralateral side. A contrast dye (Iopromide; Ultravist) was injected into the catheter while taking X-rays of the area of interest to visualize the AVF and contralateral femoral artery patency. Angiography was followed by optical coherence tomography (OPTIS OCT from St. Jude Medical) of the anastomosis and contralateral side via carotid approach to measure the inside diameter and the cross-sectional area of the vessels to delineate the vessel’s wall anatomy (intima, media, and adventitia), the presence as well as characterization of neointimal hyperplasia, thrombosis, neo-vascularization, luminal diameter, and percent diameter stenosis. For OCT, a 0.014-inch guidewire was positioned in the proximal FA and the OCT catheter (Dragonfly™ DUO imaging catheter; Abbott, Illinois, USA) was advanced over the guidewire. A nonionic, low-osmolar iodinated contrast agent, Ultravist, was simultaneously injected during OCT pullback, and the entire region of interest was scanned and images were analyzed using Light Lab OCT imaging proprietary software (Light Lab Imaging/Abbott). A comparative analysis between the AVF side femoral artery and contralateral side femoral artery was done for USG, angiography, and OCT results.

### 2.3. Histomorphology

Hematoxylin and Eosin (H&E) and Movat Pentachrome staining were done as per the standard protocol in our lab to assess the inflammation, fibrosis, vessel stenosis, and thrombosis. For H&E staining, after deparaffinization and rehydration through a series of xylene, alcohol, and distilled water, the tissue sections were stained with hematoxylin (45 s) followed by eosin (8–10 dips). The stained slides were mounted with xylene-based mounting media. For Movat Pentachrome staining, the tissue sections were deparaffinized and rehydrated and the sections were stained using a modified Russell-Movat Pentachrome kit following the manufacturer’s protocol (Cat no. KTRMPPT from American MasterTech scientific laboratory supplies). Stained tissue sections were scanned at 100 µm using a light microscope (Leica DM6). All the scanned images were blindly reviewed by two independent observers.

### 2.4. Bulk RNA Sequencing and DEGs for Inflammation

Total RNA was extracted from the collected samples (three AVF sides and three contralateral sides) using TRIZOL (Trizol reagent, Sigma, Cat# T9424, St Louis, MO, USA) following the manufacturers’ guidelines. The yields of total RNA were measured using a NanoDrop 2000 (Thermo Scientific, Waltham, MA, USA) and 1 ug of total RNA was sent (Genewiz LLC, South Plainfield, NJ, USA) for bulk RNA sequencing. The RNA samples with RIN > 6 were subjected to sequencing. The RNA samples were quantified using a Qubit 2.0 Fluorometer (ThermoFisher Scientific, Waltham, MA, USA) and RNA integrity was checked using TapeStation (Agilent Technologies, Palo Alto, CA, USA). The RNA sequencing libraries were prepared using the NEBNext Ultra II RNA Library Prep Kit for Illumina according to the manufacturer’s instructions (New England Biolabs, Ipswich, MA, USA). Briefly, mRNAs were initially enriched with Oligod(T) beads. Enriched mRNAs were fragmented for 15 min at 94 °C. First-strand and second-strand cDNA were subsequently synthesized. cDNA fragments were end-repaired and adenylated at 3′ends, and universal adapters were ligated to cDNA fragments, followed by index addition and library enrichment by PCR with limited cycles. The sequencing libraries were validated on the Agilent TapeStation (Agilent Technologies, Palo Alto, CA, USA), and quantified by using Qubit 2.0 Fluorometer (ThermoFisher Scientific, Waltham, MA, USA) as well as by quantitative PCR (KAPA Biosystems, Wilmington, MA, USA).

The sequencing libraries were multiplexed and clustered onto a flowcell. After clustering, the flowcell was loaded onto the Illumina HiSeq instrument according to the manufacturer’s instructions. The samples were sequenced using a 2 × 150 bp Paired-End (PE) configuration. Image analysis and base calling were conducted by the HiSeq Control Software (HCS). Raw sequence data (.bcl files) generated from Illumina HiSeq was converted into fastq files and de-multiplexed using Illumina bcl2fastq 2.17 software. One mismatch was allowed for index sequence identification. After investigating the quality of the raw data, sequence reads were trimmed to remove possible adapter sequences and nucleotides with poor quality using a Trimmomatic v.0.36. The trimmed reads were mapped to the Sus scrofa reference genome available on ENSEMBL using the STAR aligner v.2.5.2b. BAM files were generated because of this step. Unique gene hit counts were calculated by using feature counts from the Subread package v.1.5.2. Only the unique reads that fell within exon regions were counted. After extraction of gene hit counts, the gene hit counts table was used for downstream differential expression analysis. Using DESeq2, a comparison of gene expression between the groups of samples was performed. The Wald test was used to generate *p* values and Log_2_ fold changes. Genes with adjusted *p* values < 0.05 and absolute log_2_ fold changes >1 were called differentially expressed genes for each comparison. 

### 2.5. Quantitative Real-Time PCR

To examine the gene expression of a few selected genes, qRT-PCR was done after preparing cDNA from isolated mRNA. An iScript cDNA Synthesis Kit (BioRad # 1708891) was used to prepare cDNA and the prepared cDNA was subjected to qRT-PCR in triplicates with SYBR green (BioRad # 1725122) using a CFX96 Touch Real-Time PCR Detection System. All the primers used in this study (Table 1) were designed using NCBI (assessed on 13 December 2021) and purchased from Integrated DNA Technologies (Coralville, IA, USA). The PCR cycling conditions were 5 min at 95 °C for initial denaturation, 40 cycles of the 30 s each at 95 °C (denaturation), 30 s at 55–60 °C (depending on primer annealing temperature), and 30 s at 72 °C (extension) followed by melting curve analysis. The folds change in mRNA expression relative to controls was analyzed using 2^-^^ct^ after normalization with 18S as a housekeeping gene. 

### 2.6. Statistical Analysis

The data is presented as mean ± SD. GraphPad Prism 9 was used to analyze the RT-PCR data and the comparison between two groups for the fold change in gene expression was performed using One-way ANOVA with Bonferroni’s posthoc correction and Students’ *t*-test was used for statistical analysis. A probability (*p*) value of <0.05 was accepted as statistically significant. * *p* < 0.05, ** *p* < 0.01, *** *p* < 0.001 and **** *p* < 0.0001.

## 3. Results

### 3.1. Radiological Assessment and Histomorphology

**Figure 1:**
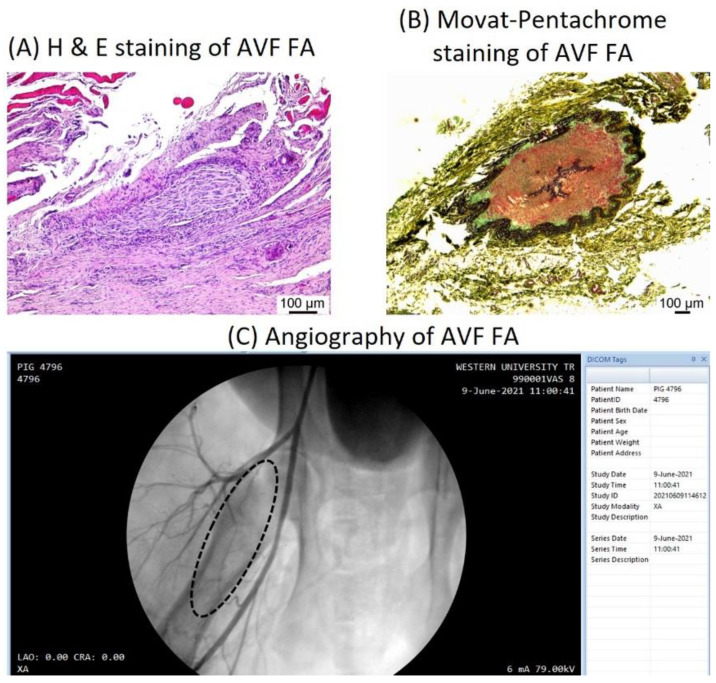
Histomorphological and radiological analysis of the femoral artery involved in the arteriovenous fistula. Hematoxylin and eosin staining (**panel A**) showed a thrombosed artery with inflammation in the adventitia. Movat-pentachrome staining (**panel B**) revealed a thrombosed artery with elastin degradation in the adventitia and increased collagen deposition in media and intima. Angiography (**panel C**) revealed a stenosed superficial femoral artery (the dotted circle) involved in AVF. These images are representative of the stenosed arteries.

Compared to the preoperative baseline assessment, post-surgical assessment of the FA involved in AVF showed partially blocked or stenosed FA with the presence of neointimal hyperplasia (NIH) and large plaques significantly obstructing the lumen of the vessel. The flow volume and velocities were decreased compared to baseline and contralateral FA. Angiography was performed for the FA in which guidewire can be advanced to the site of AVF. Angiography showed open FA in some swine while others, mainly in scrambled and vehicle groups, showed thrombosed and stenosed arteries. In FA showing no flow in doppler ultrasound, a guidewire could not be advanced, and no angiography was done. Similarly, OCT was performed only in the arteries which were open and not for the blocked arteries. Open arteries showed normal OCT while OCT was not done in thrombosed arteries due to technical difficulty in advancing the guidewire. The H&E staining and Movat-Pentachrome staining also showed blocked FA and extensive fibrosis around vessels at the AVF site (Figure 1).

### 3.2. DEGs Related to Inflammation

Bulk RNA sequencing of the tissues revealed a total of 14,554 genes, of which 415 were significantly expressed genes (*p* < 0.05 and log_2_ fold > 1). From the list of differentially expressed genes (DEGs) with *p* < 0.05 and log_2_ fold > 1, genes with *p*-value < 0.05 and log_2_ fold > 2 were sorted out (Supplementary Material 1). An extensive literature search was done to check all DEGs for their role in inflammation in any organ system of the body; migration, proliferation, and activation of immune cells including macrophages, neutrophils, natural killer (NK) cells, B- and T-lymphocytes, T-regulatory (Treg) cells, and dendritic cells (DCs); activation and regulation of innate and adaptive immune response; and regulation of cytoplasmic kinases including nuclear factor-kappa beta (NF-κB), phosphoinositide 3-kinases (PI3K), protein kinase B (PKB/Akt), and mitogen-activated protein kinase (MAPK). These parameters were included in the literature search because of their crucial role in regulating inflammation and inflammatory signaling. We found various DEGs (Table 2, Table 3, Table 4 and Table 5) playing a crucial role in inflammation, regulation of inflammatory pathways, and immune regulation. Among the DEGs involved in inflammation, some of the genes which were differentially expressed (positive log_2_ fold value) in scrambled peptide treated FA were found downregulated (negative log_2_ fold values) after treatment with TREM-1 inhibitor (LR12) + TLR-4 inhibitor (TAK-242) suggesting the effect of attenuating inflammation by inhibiting TREM-1 and TLR-4. Additionally, the gene occurring in different comparison groups was included only in one table and not in others (the details of all DEGs involved in inflammation and immune regulation in each group can be found in Supplementary Material 1). 

### 3.3. Quantitative Real-Time PCR

**Figure 2:**
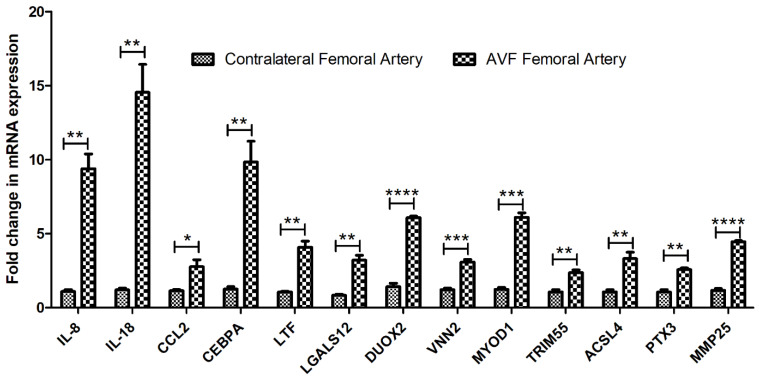
RT-PCR for mRNA expression of DEGs in femoral artery involved in arteriovenous fistula compared to the contralateral femoral artery. All data are presented as mean ± standard deviation (SD). A *p*-value < 0.05 was considered significant. * *p* < 0.05, ** *p* < 0.01, *** *p* < 0.001, **** *p* < 0.0001. Interleukin (IL), C-C Motif Chemokine Ligand 2 (CCL2), CCAAT Enhancer Binding Protein Alpha (CEBPA), Lactotransferrin (LTF), Galectin 12 (LGALS12), Dual Oxidase 2 (DUOX2), Vanin 2 (VNN2), Myoblast Determination Protein 1 (MYOD1), Tripartite Motif Containing 55 (TRIM55), Pentraxin 3 (PTX3), Matrix metalloproteinase 25 (MMP25).

Quantitative RT-PCR revealed increased expression of IL-8, IL-18, MYOD1, CEBPA, LGALS12, TRIM55, ACSL4, CCL2, DUOX2, PTX3, MMP25, LTF, and VNN2 in FA involved in AVF compared to contralateral FA (Figure 2). Significantly increased expression of these genes in AVF FA suggests the presence of inflammation and supports the findings of RNA sequencing data with higher expression of these DEGs in AVF FA compared to contralateral FA.

### 3.4. Network Analysis

**Figure 3:**
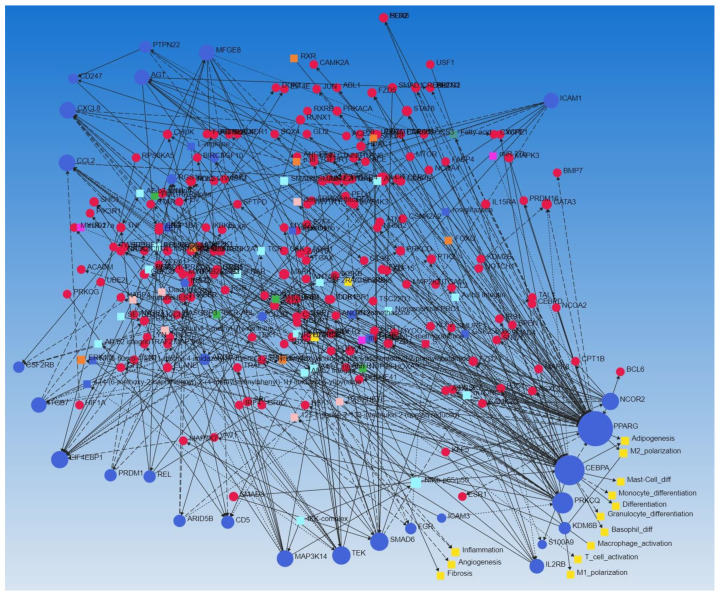
Signor network analysis of the DEGs. The network analysis showed the correlation of DEGs with each other. Blue circles show the DEGs listed in Table 2, Table 3, Table 4 and Table 5 and involved in inflammation, immune response, and remodeling (CXCL8, CCL2, ICAM1, ICAM3, ITGB7, PPARG, REL, IL2, S100A9, etc.), yellow circles show cellular mechanisms involved in chronic inflammation and plaque formation including macrophage polarization, fibrosis, inflammation, angiogenesis, and lymphocytes activation.

**Figure 4:**
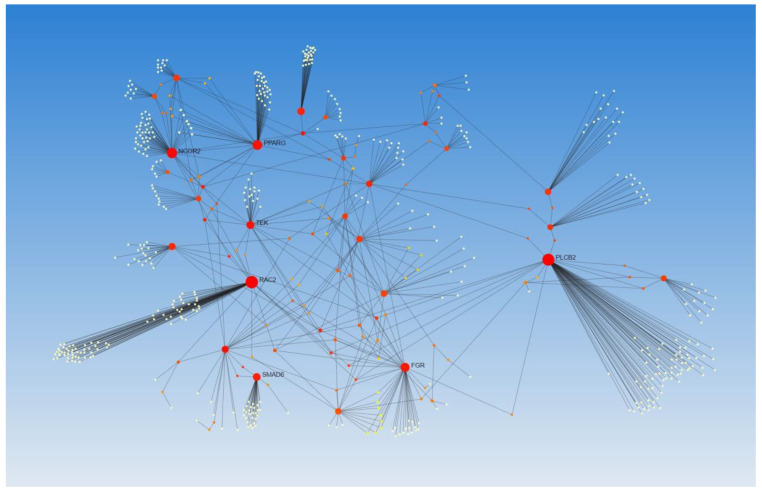
STRING analysis for protein-protein interaction. The analysis showed that various DEGs are related to each other and regulate each other.

The network analysis (Signor for regulatory network analysis and STRING for protein-protein interaction networkanalyst.ca) using the selected DEGs as input showed the regulatory interaction of these genes with each other and the role of these DEGs in inflammation, angiogenesis, and fibrosis, in addition to the three contributing factors in vessel thrombosis (Figure 3 and Figure 4). The network analysis also revealed the role of these DEGs in M1 and M2 macrophages polarization, T-cell activation, macrophage activation, basophils, granulocytes, and monocytes differentiation. The increased expression of these DEGs in FA involved in AVF and their association with inflammation, regulation of inflammation, immune cell differentiation, and activation suggest the role of these DEGs in chronic inflammation which precipitates vascular thrombosis.

## 4. Discussion

Chronic inflammation and inflammatory immune cells play a critical role in the development and progression of atheromatous plaque formation contributing to thrombosis and vessel stenosis. The development and progression of atherosclerosis are characterized by deposition of low-density lipoproteins in vessel intima, formation of a fatty streak, foam cell formation, plaque formation, and immune cell infiltration followed by thrombosis and vessel stenosis. Persistent infiltration of pro-inflammatory immune cells causing increased secretion of pro-inflammatory cytokines and increased expression of inflammatory mediators leads to chronic inflammation contributing to thrombosis [11,15,16,17,18,19]. Thus, targeting inflammation seems a promising therapeutic strategy to attenuate the progression of atheromatous plaque and thrombosis. Thrombosis of the vessels participating in AVF is a common cause to precipitate early AVF failure [20,21]. Since inflammation plays a crucial role in vessel thrombosis and studies have shown the crucial role of inflammation-intimal hyperplasia-plaque-thrombosis, we focused on investigating the factors mediating adventitial inflammation and inflammation of the surrounding structure possibly playing a role in early vessel thrombosis and AVF failure. In other words, the focus of this study was to focus on the role of inflammation in the vicinity of AVF. The inflammation may either be due to surgical injury or the immune response of the body. Inflammation of the vessel adventitia, around a blood vessel and vicinity tissue, causes perivascular cuffing due to the accumulation of immune cells [22,23] and contributes to vessel stenosis [10]. 

**Figure 5:**
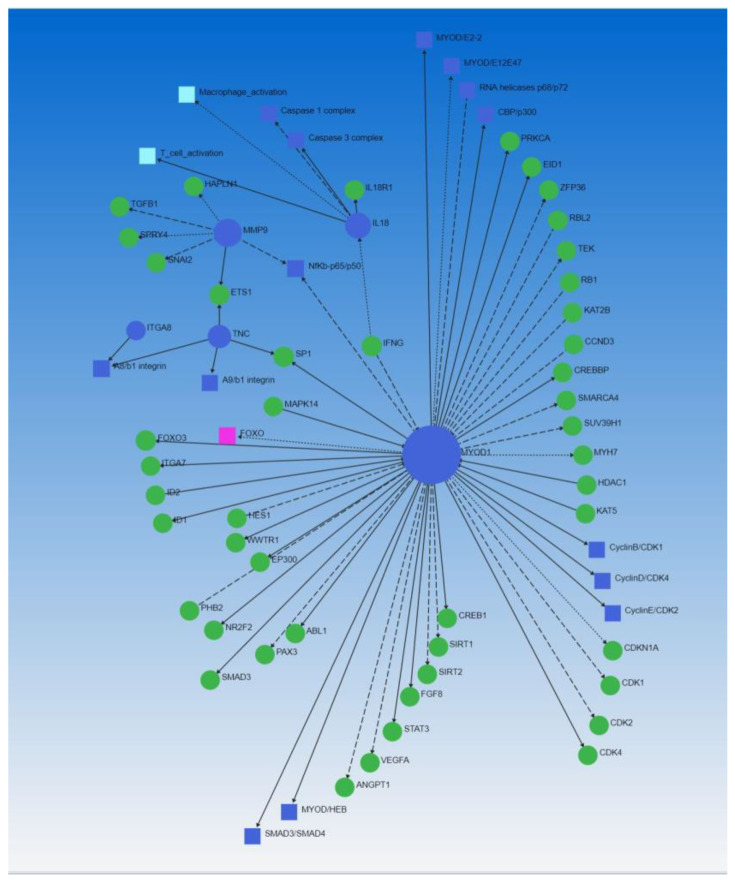
MYOD1 regulates the expression of various DEGs involved in chronic inflammation, plaque formation, and thrombosis.

A significantly increased TRIM55 (Murf2) expression in AVF FA tissue with RNA seq (log_2_ fold = 8.1, Table 2) suggests that increased expression of TRIM55 might have caused perivascular cuffing of the FA and chronic inflammatory milieu precipitating vessel thrombosis [24]. Increased TRIM55 might be in response to muscle injury while creating AVF or post-surgical muscle atrophy [25,26]. An association of increased perivascular cuffing with increased TRIM55 expression and decreased infiltration of immune cells and perivascular cuffing with TRIM55 knock out as reported previously [27,28] suggests TRIM55 as a potential therapeutic target to attenuate perivascular cuffing and an increased TRIM55 expression in AVF FA compared to contralateral FA suggests a chronic inflammatory state around the AVF FA mediating vessel thrombosis and stenosis. Another DEG that was significantly increased in AVF FA compared to control FA was myogenic differentiation 1 (MYOD1) (log_2_ fold = 7.29). MYOD1 is a transcription factor that regulates muscle differentiation [29], and its expression varies with the presence and absence of inflammation [30,31]. Although MYOD1 is associated with muscle differentiation, its association with inflammation coerced us to investigate the regulatory network of MYOD1. The gene regulatory network using all 425 DEGs revealed MYOD1 association with nuclear factor kappa beta (NF-κB), interferon-gamma (IFN-γ), integrins, interleukin (IL)-18, caspases, matrix metalloproteinases (MMPs), sirtuins, vascular endothelial growth factor A (VEGFA), angiopoietin 1 (ANGPT1), and immune cell activation (Figure 5). The association of MYOD1 with the genes involved in inflammation, angiogenesis, arteriogenesis, and immune cell activation suggests its probable role in the pathologies involved in vessel thrombosis (inflammation, immune cell activation, and angiogenesis). Increased expression of MYOD1 in association with other genes involved in inflammation namely IL-8 (CXCL8, log_2_ fold = 2.21), IL-18 (log_2_ fold = 2.84), CCR7 (log_2_ fold = 5.30), ITGB7 (log_2_ fold = 3.85), MMP-9 (log_2_ fold = 5.58), MMP-25 (log_2_ fold = 4.14), and TREML1 (log_2_ fold = 5.11) suggest the role of MYOD1 in perivascular cuffing. Furthermore, an association of these DEGs with thrombosed AVF FA suggests the role of these DEGs in perivascular cuffing and vessel thrombosis.

Tissue injury is associated with increased secretion of S100 proteins. The S100 proteins S100A8, S100A9, and S100A12 play a crucial role in inflammation and atherosclerosis [16,32], while S100A1 plays a role in post-ischemic angiogenesis [33]. S100 proteins are potential therapeutic targets in atherosclerosis [32,34]. An increased expression of DEGs S100A1 (log_2_ fold = 3.69), S100A8 (log_2_ fold = 4.48), and S100A9 (log_2_ fold = 2.91) in RNA seq analysis of the FA involved in AVF compared to contralateral FA (Table 2, Table 3, Table 4 and Table 5) suggest the pathologic role of calgranulins in vessel thrombosis and early AVF failure. Increased expression of S100 proteins is associated with inflammation, increased secretion of proinflammatory cytokines (IL-6, IL-8, and IL-18), and immune cell infiltration (macrophages) [35,36]. Increased expression of IL-8 (CXCL8, log_2_ fold = 2.21), IL-18 (log_2_ fold = 2.84), and CCR7 (log_2_ fold = 5.30) in association with S100 proteins suggest that IL-8 and IL-18 play a critical role in vessel thrombosis. This notion is supported by the role of IL-8 secreted from macrophages in the pathogenesis of atherosclerosis [37,38,39]. Similarly, the increased expression of IL-18 is associated with atherosclerosis, and it enhances atherosclerosis in association with IFN-γ [40,41,42]. An increased expression of CCR7 (log_2_ fold = 5.30, M1 macrophage marker) and association of MYOD1 with IFN-γ suggest a possible role of IL-8 and IL-18 in vascular thrombosis, an underlying pathology for early AVF failure. Another DEG related to inflammation and involved in angiogenesis was TEK [43,44,45] whose expression was increased in AVF FA (log_2_ fold = 2.22). The TEK gene is also known as Tie2 and is an angiopoietin receptor and is involved in neoangiogenesis, which contributes to the progression and stabilization of the plaques. Tie-2 plays a key role in vessel stabilization and destabilization in association with Ang-I mediated Tie-2 activation and Ang-II mediated inhibition of Tie-2 activation [46]. Increased TEK expression in AVF FA might be due to the locally active renin-angiotensin system in the vessel intima [47], as TEK expression is regulated by Ang-I and Ang-II. The involvement of TEK in angiogenesis and inflammation supports the hypothesis of its involvement in chronic inflammation and a probable role in vessel thrombosis. This is also supported by the fact that inhibition of TEK alleviates the release of inflammatory cytokines [44]. Another DEG with increased expression was bridging integrator 2 (BIN2) (log_2_ fold = 2.63). BIN2 regulates platelet activation in thrombosis, thrombo-inflammation, and atherosclerosis, and depletion of BIN2 is associated with protection from arterial thrombosis [48,49]. Increased expression of BIN2 in AVF FA samples in this study suggests a possible role of BIN2 in early vessel thrombosis and its contribution to early AVF failure. Another DEG phospholipase C-β2 (PLCB2) was found increased (log_2_ fold = 2.66) in AVF FA tissue. PLCB2 expression is regulated by NF-κB and is involved in platelet activation, inflammation, and atherosclerosis [50]. The involvement of PLCB2 in inflammation and atherosclerosis and its increased expression in AVF FA indicates the role of PLCB2 in vessel thrombosis and probably early AVF failure. Another DEG, ABL2 (log_2_ fold = 4.69) regulates vascular leakage during inflammation, and depletion of Arg/Abl2 associates with improvement in endothelial cell adhesion and prevents vascular leakage during inflammation [51]. LGALS12 (galectin-12, log_2_ fold = 5.41) enhances inflammation by promoting M1 macrophage polarization and negatively regulates M2 macrophage polarization [52]. Increased expression of these and multiple other DEGs (Table 2, Table 3, Table 4 and Table 5) in AVF FA suggest the probable critical role of these DEGs in vessel thrombosis; however, this warrants future detailed mechanistic studies. 

Along with the various DEGs involved and inducing inflammation, we also found various DEGs with an anti-inflammatory and antiatherosclerosis function. The DEGs were OSR1 (log_2_ fold= −2.48), FFAR4 (log_2_ fold = 4.80), CEBPA (log_2_ fold = 4.06), PON1 (log_2_ fold = 3.77), MLXIPL (log_2_ fold = 3.37), HCAR1 (log_2_ fold = 2.00), GPR39 (log_2_ fold = −3.07), MFGE8 (log_2_ fold = −2.53), A4GNT (log_2_ fold = 7.78), ABCC8 (log_2_ fold = 5.78), CD5 (log_2_ fold = 4.01), and ARID5B (log_2_ fold = 2.15). Odd-skipped related transcription factor 1 (OSR1), also known as oxidative stress-responsive kinase 1 (OSXR1), inhibits NF-κB [53] and regulates hepatic inflammation [54]. Free Fatty Acid Receptor 4 (FFAR4, GPR120) has anti-atherosclerotic potential and attenuates M1 macrophage activity, thus providing anti-inflammatory activity [55]. CCAAT/enhancer-binding protein alpha (CEBPA), gene encoding C/EBPα, plays a crucial role in myeloid lineage maturation and is expressed during the late phase of inflammatory responses. A decreased secretion of inflammatory cytokines TNF-α, IL-6, IL-1β, and IFN-γ with MTL-CEBPA, a small activating RNA targeting for upregulation of C/EBPα, suggests an anti-inflammatory role of CEBPA [56]. Paraoxonase 1 (PON1) protects against lipid oxidation and has an antioxidant and anti-inflammatory role in atherosclerosis [57]. The increased expression of MLXIPL by c-Jun inhibits inflammation in spinal cord nerve injury [58]. The increased expression of hydroxycarboxylic acid receptor 1 (HCAR1) is associated with anti-inflammatory response in glaucoma [59]. Under inflammatory conditions, G protein-coupled receptor 39 (GPR39) plays an anti-inflammatory role by enhancing IL-10 production from macrophages [60]. The increased expression of milk fat globule epidermal growth factor VIII (MFGE8) is associated with aging, atherosclerosis, hypertension, and diabetic arterial walls, and plays a crucial role in remodeling [61]. MFGE8 also has an anti-inflammatory response, and treatment with recombinant MFGE8 suppresses inflammation in mouse [62]. A4GNT may protect against inflammation-associated gastric adenocarcinoma (https://www.uniprot.org/uniprot/Q14BT6; accessed on 8 January 2022) and knocking out A4GNT is associated with gastric mucosal hyperplasia [63]. ATP-binding cassette transporter sub-family C member (ABCC) 8 encodes for sulfonylurea receptor 1 (Sur1) and silencing of Abcc8 or inhibition of Sur1-Trpm4 attenuate inflammation and disease progression in experimental autoimmune encephalomyelitis. This suggests the anti-inflammatory effect of silencing ABCC8 [64]. IL-10 is an anti-inflammatory cytokine secreted by CD5+ B cells [65], and increased expression of CD5 in AVF FA samples suggests the immune response of the body to increase IL-10 secretion. IL-10 is important as it protects against atherosclerosis by regulating atherogenic macrophage function [66] and can mitigate atherosclerosis [67,68]. Since neointimal hyperplasia and progressive plaque formation contribute to vessel thrombosis, increased CD5 expression in these samples suggests a protective mechanism. The AT-Rich Interaction Domain 5B (ARID5B) gene encodes a member of the AT-rich interaction domain (ARID) family of DNA binding proteins and methylation of ARID5B prevents inflammation and progression and development of atherosclerosis by inhibiting the ox-LDL/PI3K/Akt/NF-κB pathway [69].

The presence of DEGs contributing to pro-and anti-inflammatory pathogenesis in AVF FA samples compared to contralateral FA indicate the presence of chronic inflammation in AVF tissue as well as the anti-inflammatory immune response of the body. The presence of both pro-and anti-inflammatory DEGs suggests an immune response of the body to attenuate chronic inflammation but the outnumbering of the number of pro-inflammatory DEGs compared to the number of anti-inflammatory DEGs suggests the presence of persistent chronic inflammation. The presence of inflammatory DEGs and persistent inflammation might be the cause of early vessel thrombosis and early AVF failure. Furthermore, the presence of DEGs involved in perivascular cuffing suggests that for AVF patency, targeting inflammation around the vessel along with the pathologies inside the lumen (neointimal hyperplasia, intimal inflammation, plaque formation) should be considered. Targeting inflammation and immune cells to attenuate atherosclerosis supports the notion of targeting perivascular cuffing and luminal inflammation to enhance AVF maturation [19]. Targeting T-cells and macrophage accumulation to regulate adaptive venous remodeling increase AVF maturation [70], and increased expression of CCR7 (log_2_ fold = 5.30), a marker for the pro-inflammatory M1 macrophage, indicate the significance of targeting inflammatory macrophages for AVF maturation. Adaptive vascular extracellular matrix (ECM) remodeling favors outward remodeling of the vessel and contributes to AVF maturation. Thus, not only adaptive intimal ECM remodeling but adventitial remodeling also contributes to AVF maturation. Collagen deposition and elastin degradation play a crucial role in ECM remodeling, which is an attractive target for AVF maturation [71]. The presence of various DEGs associated with ECM remodeling including ELN, ITGA8, PRELP, FMOD, ITGA3, ADAMTSL4, HSPE, OSMR, CHST2, ECM1, and matrix metalloproteinases including MMP9, MMP7, MMP25, MMP17, and MMP8, and in our data indicate the probability of targeting vascular adventitial and ECM remodeling for AVF maturation. Targeting MMPs and ECM remodeling in association with inflammation is important because MMP activity is regulated by inflammatory cytokines and MMPs regulate inflammatory processes [72,73,74]. Targeting inflammation to enhance outward remodeling is also supported by favorable remodeling in mice deficient in the TLR-4 homolog RP105 [75].

## 5. Targeting DEGs and Translational Aspect

Chronic inflammation critically contributes to vessel thrombosis and stenosis. The presence of inflammatory DEGs and their correlation with inflammatory processes in this study support the notion of targeting these DEGs to attenuate chronic inflammation. Furthermore, measuring the serum expression of these DEGs may be used as biomarkers of the ongoing thrombosis and stenosis of the femoral vessels and AVF, as increased expression of these DEGs indicate thrombosis and an ongoing AVF failure. Thus, a timely intervention may be taken. Further, the presence of significantly increased inflammatory DEGs is also indicative of the ongoing inflammation and the need for anti-inflammatory treatment targeting these genes after AVF creation. The presence of thrombosed arteries also suggests the possibility of anti-platelet or anti-thrombotic treatment before and after AVF creation to attenuate plaque formation and early thrombosis [76,77].

Additionally, various DEGs delineated in this analysis involved in chronic inflammation also regulate other cellular mechanisms including fibrosis, ECM remodeling, vascular smooth muscle cell proliferation, and phenotype change (Figure 5, MYOD1 regulates many genes and TFs). These changes can be investigated using high-resolution scanning of the AVF, such as high-resolution 3D imaging [78]. High-resolution imaging will help in assessing AVF at various time points and an evolving thrombus or vessel stenosis can be evaluated early before complete occlusion. This will enhance the clinical outcome and may also help maintain AVF patency for a longer time. However, these assumptions warrant well-organized large-scale clinical trials. 

## 6. Conclusions

Overall, the RNA sequencing results of AVF FA and contralateral control FA revealed multiple DEGs involved in inflammation, inflammatory pathways, immune cell migration, and proliferation and regulation of cytoplasmic kinases. Additionally, the increased expression of genes related to skeletal muscle injury and playing a role in vascular cuffing support the notion of targeting chronic inflammation in the vicinity of AVF along with the luminal pathologies. The tissues in this study were treated with the inhibitors of TREM-1 and TLR-4 to attenuate plaque formation by decreasing inflammation; however, the presence of various DEGs related to inflammation indicates a more aggressive approach to attenuate inflammation due to skeletal muscle injury and target perivascular cuffing, adventitial inflammation, and remodeling.

## 7. Limitations of the Study

This study elucidated several significantly expressed DEGs involved in inflammation, inflammatory pathogenesis, and the regulation of innate and adaptive immune response and proposed targeting chronic inflammation in the perivascular space due to vessel and skeletal muscle injury which might have therapeutic potential in AVF maturation. The limited number of samples and treatment with TREM-1 and TLR-4 inhibitors might have confounded the outcome. Furthermore, the involvement of several DEGs in inflammation as discussed here has not been shown in the perspective of atherosclerosis, and warrants future in vitro and in vivo studies. A comparison of AVF tissue without any treatment with treated tissues may reveal more DEGs involved in inflammation and as a therapeutic target. Despite these limitations, this study elucidated novel DEGs that can be targeted to attenuate or inhibit early vascular thrombosis to hasten AVF maturation. The focus on vascular adventitia remodeling and attenuating perivascular cuffing by targeting DEGs involved in inflammation is a key point of this manuscript.

## Figures and Tables

**Table 1:** The nucleotide sequence (5′--3′) of the primers used for a real-time quantitative polymerase chain reaction. Interleukin (IL), C-C Motif Chemokine Ligand 2 (CCL2), CCAAT Enhancer Binding Protein Alpha (CEBPA), Lactotransferrin (LTF), Galectin 12 (LGALS12), Dual Oxidase 2 (DUOX2), Vanin 2 (VNN2), Myoblast Determination Protein 1 (MYOD1), Tripartite Motif Containing 55 (TRIM55), Pentraxin 3 (PTX3), Matrix metalloproteinase 25 (MMP25).

Gene Name	Forward Primer	Reverse Primer
IL-8	5′-GACCCCAAGGAAAAGTGGGT-3′	5′-TGACCAGCACAGGAATGAGG-3′
IL-18	5′-ATGGCTGCTGAACCGGAAG-3′	5′-GGTCTTCATCGTTTTCAGCTACA-3′
MYOD1	5′-GCTCCGCGACGTAGATTTGA-3′	5′-GGAGTCGAAACACGGGTCAT-3′
CEBPA	5′-CGGTGCGTCTAAGATGAGGG-3′	5′-AGGCACATATTTGCTCCCCC-3′
LGALS12	5′-GACCCGCTTCCTGACACCTT-3′	5′-CCCTCCACAAACGGGTTGAT-3′
TRIM55	5′-CGTAGGGCCTTCAGGTTCTG-3′	5′-GTTCCCTTACCACTCACCGC-3′
ACSL4	5′-TCTGTTCGCTGTTGCTGATTTG-3′	5′-GAGAGCCCGCCACACAAGT-3′
CCL2	5′-AAACGGAGACTTGGGCACAT-3′	5′-CTTGCAAGGACCCTTCCGTC-3′
DUOX2	5′-GCTCTGCATAAGACCAGAGGC-3′	5′-GTCAGTGAGAGTGCGTCCTG-3′
PTX3	5′-GCAGGTTGTGAAACAGCGAT-3′	5′-TTTGACCCAAATGCAGGCAC-3′
MMP25	5′-TTGTCCTGGCGTTTCTGTGT-3′	5′-GGCCATCAGCTTGGCTCATA-3′
LTF	5′-GTCACAGCCATCGCTAACCT-3′	5′-TTGCTCCTCCAAGCTTGACCT-3′
VNN2	5′-GATGTCCCTGAAAAGCCGGA-3′	5′-GTCACAGCAGGATCACGGAA-3′

**Table 2:** Differentially expressed genes (DEGs) while comparing contralateral femoral artery with AVF femoral artery combining all tissues (+value = higher expression in AVF FA; −value = higher expression in contralateral FA).

Gene ID	log_2_ FoldChange	*p*-Value	Gene Name
ENSSSCG00000006216	8.11	0.00000000772	TRIM55
ENSSSCG00000007436	5.58	0.0000000953	MMP-9
ENSSSCG00000006590	4.48	0.00000979	S100A8
ENSSSCG00000009645	3.96	0.0000292	ADAMDEC1
ENSSSCG00000031053	3.69	0.000000542	S100A1
ENSSSCG00000022512	3.20	0.0000546	TRDC
ENSSSCG00000023842	3.05	0.000389967	TRAT1
ENSSSCG00000028331	3.01	0.000191823	IL1R2
ENSSSCG00000015037	2.84	0.0000120	IL-18
ENSSSCG00000006309	2.78	0.000659109	CD247
ENSSSCG00000006025	2.71	0.000426117	PKHD1L1
ENSSSCG00000014310	2.58	0.000507304	CXCL14
ENSSSCG00000006452	2.31	0.000310425	CD1D
ENSSSCG00000003113	2.08	0.000494243	C5AR2
ENSSSCG00000009051	2.04	0.000959894	IL-15
ENSSSCG00000008606	−2.48	0.000000136	OSR1

**Table 3:** Differentially expressed genes (DEGs) while comparing contralateral FA with LR-12 + TAK-242 treated AVF FA (+value = higher expression in AVF FA; −value = higher expression in contralateral FA).

Gene ID	log_2_ FoldChange	*p*-Value	Gene Name
ENSSSCG00000022490	7.12	0.037585934	GPR83
ENSSSCG00000016688	7.06	0.040958471	CPVL
ENSSSCG00000013056	5.41	0.000202896	LGALS12
ENSSSCG00000010478	4.80	0.000919971	FFAR4
ENSSSCG00000016878	4.17	0.011959826	FGF10
ENSSSCG00000015015	4.16	0.008270436	ARHGAP20
ENSSSCG00000002866	4.05	0.000885129	CEBPA
ENSSSCG00000011579	4.04	0.00094779	PPARG
ENSSSCG00000015332	3.77	0.041851053	PON1
ENSSSCG00000026297	3.76	0.00389716	KLB
ENSSSCG00000008237	3.50	0.002121156	RETSAT
ENSSSCG00000007710	3.37	0.005462755	MLXIPL
ENSSSCG00000011831	3.21	0.003915618	APOD
ENSSSCG00000015267	2.77	0.010298314	FMO2
ENSSSCG00000017705	2.61	0.014997339	CCL5
ENSSSCG00000014880	2.23	0.045711441	AQP11
ENSSSCG00000005122	2.22	0.020695275	TEK
ENSSSCG00000008953	2.21	0.031773067	CXCL8
ENSSSCG00000025578	2.19	0.026455299	ALDH1A2
ENSSSCG00000003578	2.17	0.032743557	FGR
ENSSSCG00000008624	2.16	0.024212176	LPIN1
ENSSSCG00000029813	2.10	0.025936426	TSPAN5
ENSSSCG00000013886	2.07	0.038238135	B3GNT3
ENSSSCG00000009789	2.00	0.044237292	HCAR1
ENSSSCG00000000211	−3.42	0.017397621	AQP5
ENSSSCG00000015707	−3.07	0.192328785	GPR39
ENSSSCG00000008835	−2.84	0.007206502	RASL11B
ENSSSCG00000001834	−2.53	0.011049962	MFGE8

**Table 4:** Differentially expressed genes (DEGs) while comparing contralateral FA with scrambled peptide treated AVF FA (+value = higher expression in AVF FA treated with scrambled peptide; −value = higher expression in contralateral FA).

Gene ID	log_2_ FoldChange	*p*-Value	Gene Name
ENSSSCG00000005951	9.28	0.002454177	TMEM71
ENSSSCG00000011862	8.97	0.004185163	MUC13
ENSSSCG00000029879	8.17	0.016038726	LTF
ENSSSCG00000015748	7.92	0.023568215	CSMD1
ENSSSCG00000022473	7.78	0.029138305	A4GNT
ENSSSCG00000008972	7.45	0.046820249	PPEF2
ENSSSCG00000027568	6.64	0.000784677	BLK
ENSSSCG00000025042	6.02	0.007096705	ICOS
ENSSSCG00000013378	5.78	0.041425329	ABCC8
ENSSSCG00000011131	5.58	0.016369542	PRKCQ
ENSSSCG00000017466	5.30	0.002770819	CCR7
ENSSSCG00000001613	5.11	0.001355889	TREML1
ENSSSCG00000006266	5.01	0.022187352	ST18
ENSSSCG00000002821	4.88	0.008741294	CCL22
ENSSSCG00000006734	4.68	0.005857569	CD101
ENSSSCG00000029668	4.52	0.011344171	IL2RB
ENSSSCG00000015093	4.50	0.004868833	CD3D
ENSSSCG00000021569	4.14	0.002023038	MMP25
ENSSSCG00000013115	4.01	0.00956326	CD5
ENSSSCG00000000257	3.85	0.004632273	ITGB7
ENSSSCG00000004195	3.84	0.004380652	ARG1
ENSSSCG00000030042	3.76	0.002440454	SBNO2
ENSSSCG00000006359	3.57	0.003335966	ADAMTS4
ENSSSCG00000017962	3.49	0.004592719	KDM6B
ENSSSCG00000000705	3.49	0.027859902	CD27
ENSSSCG00000009630	3.48	0.008741686	EGR3
ENSSSCG00000000605	3.46	0.036481122	ERP27
ENSSSCG00000013649	3.40	0.01209607	ICAM3
ENSSSCG00000013839	3.36	0.043788189	RASAL3
ENSSSCG00000015550	3.15	0.045746825	RGS16
ENSSSCG00000000136	3.10	0.009086041	CSF2RB
ENSSSCG00000000688	3.07	0.02332297	LAG3
ENSSSCG00000014825	3.06	0.016131597	RELT
ENSSSCG00000004678	3.05	0.009097692	DUOX2
ENSSSCG00000011727	2.93	0.011434284	PTX3
ENSSSCG00000006588	2.91	0.044677952	S100A9
ENSSSCG00000003805	2.83	0.013758246	PDE4B
ENSSSCG00000000521	2.83	0.012411864	PHLDA1
ENSSSCG00000015299	2.79	0.028194104	STEAP4
ENSSSCG00000004179	2.69	0.028039502	VNN2
ENSSSCG00000006379	2.67	0.029518927	CD48
ENSSSCG00000004779	2.66	0.037184423	PLCB2
ENSSSCG00000008388	2.64	0.044215498	REL
ENSSSCG00000021944	2.63	0.025402199	RAC2
ENSSSCG00000000223	2.63	0.043774003	BIN2
ENSSSCG00000011443	2.62	0.018907549	STAB1
ENSSSCG00000006800	2.41	0.02415221	CD53
ENSSSCG00000013655	2.29	0.034717888	ICAM1
ENSSSCG00000017330	2.17	0.034875529	MAP3K14
ENSSSCG00000010219	2.15	0.033546298	ARID5B
ENSSSCG00000012583	2.10	0.038092469	ACSL4
ENSSSCG00000017723	2.09	0.038906102	CCL2
ENSSSCG00000006002	−2.12	0.035313216	NOV
ENSSSCG00000024259	−3.50	0.024778737	PPP1R11
ENSSSCG00000004948	−2.33	0.030111699	SMAD6

**Table 5:** Differentially expressed genes (DEGs) while comparing scrambled peptide treated FA with LR-12 + TAK-242 treated AVF FA (+value = higher expression in scrambled peptide treated AVF FA; −value = higher expression in LR-12 + TAK-242 treated AVF femoral artery).

Gene ID	log_2_ FoldChange	*p*-Value	Gene Name
ENSSSCG00000007717	3.57	0.022045439	METTL27
ENSSSCG00000013056	3.53	0.007938274	LGALS12
ENSSSCG00000010184	3.33	0.007715639	AGT
ENSSSCG00000003399	3.27	0.021031658	RBP7
ENSSSCG00000012667	3.12	0.014533603	IGSF1
ENSSSCG00000028996	3.05	0.013185638	ALDH1A1
ENSSSCG00000029813	2.74	0.019674447	TSPAN5
ENSSSCG00000000576	2.69	0.019345908	LDHB
ENSSSCG00000020657	2.55	0.024205833	BCAM
ENSSSCG00000013260	2.45	0.031013151	MDK
ENSSSCG00000015249	2.40	0.036414276	ADAMTS8
ENSSSCG00000009492	2.34	0.040360908	GPR180
ENSSSCG00000017605	2.34	0.036679735	MMD
ENSSSCG00000016331	2.34	0.035784764	RAMP1
ENSSSCG00000022301	2.19	0.047966521	EIF4EBP1
ENSSSCG00000006764	−7.32	0.032418272	PTPN22
ENSSSCG00000005236	−7.12	0.043128193	DMRT1
ENSSSCG00000007956	−5.37	0.047045343	NLRC3
ENSSSCG00000007523	−5.15	0.000483159	TUBB1
ENSSSCG00000015656	−4.58	0.028406123	FCMR
ENSSSCG00000010772	−4.16	0.002898421	ADAM8
ENSSSCG00000009237	−3.74	0.00903231	HPSE
ENSSSCG00000006378	−3.73	0.007424014	SLAMF7
ENSSSCG00000000653	−3.68	0.007197867	CD69
ENSSSCG00000017908	−3.66	0.036598715	GP1BA
ENSSSCG00000013853	−3.52	0.007956036	HSH2D
ENSSSCG00000010575	−3.20	0.009548539	PPRC1
ENSSSCG00000004369	−2.86	0.034230241	PRDM1
ENSSSCG00000013041	−2.38	0.034963931	FERMT3
ENSSSCG00000009761	−2.30	0.036755714	NCOR2
ENSSSCG00000017333	−2.27	0.041076061	FMNL1

## Data Availability

All data supporting the results of this manuscript has been included in this manuscript along with a Supplementary File. Bulk RNA seq (Fastq. Files) can be provided from the corresponding authors on request.

## Supplementary Materials

The following supporting information can be downloaded at: https://www.mdpi.com/article/10.3390/biomedicines10020433/s1, Table S1: Differentially Expressed Genes (DEGs) in various groups.
